# Cambodia's disconnection from ASEAN: a social disorganization theory's interpretation

**DOI:** 10.3389/fsoc.2025.1529649

**Published:** 2025-08-05

**Authors:** Bama Andika Putra

**Affiliations:** ^1^School of Sociology, Politics, and International Studies, University of Bristol, Bristol, United Kingdom; ^2^Department of International Relations, Universitas Hasanuddin, Makassar, Indonesia

**Keywords:** regional organizations, social disorganization theory, sociological approach, social sciences, Cambodia

## 1 Cambodia and the ASEAN way: recent trends

Established in 1967, the Association of Southeast Asian Nations (ASEAN) is a regional organization that upholds the values of non-interference, non-intervention, and consensus-based decision-making (Acharya, 2021). Scholars and observers have termed those values collectively as the “ASEAN Way” (Caballero-Anthony, 2005; Chongkittavorn, 2023; Putra, 2024b; Ravenhill, 2007; Wahyuningrum, 2014). It is the belief that despite the great power political rivalries taking place in the region, the organization would continue to be relevant in guiding its member states to remain neutral and press down negative implications of those contestations (Acharya, 1998, 2014, 2019; Anwar, 2020; Darwis et al., 2020). However, it does so in a way that does not confer supranational authority on the regional organization, as is the case with the European Union and other regional organizations. Doing so has secured the allegiances of ASEAN member states to abide by the ASEAN Way voluntarily, allowing for the continued relevance of ASEAN in regional politics for decades. Nevertheless, with the challenges encountered evolving to include great powers exerting their influence in Southeast Asia, have member states remained true to the ASEAN Way?

Nevertheless, the rise of China in the Asia-Pacific region has presented some dilemmas for Southeast Asian states. On the one hand, they perceive China's rise as an opportunity, as China's Belt and Road Initiative (BRI)—related funding could assist the development of Southeast Asian states (Hu, 2019; World Bank, 2018; Yu, 2024). Nevertheless, they are also aware that China's rise has been accompanied by its growing assertiveness in the political and security domains, such as China's power projections in the South China Sea (Collin, 2016; Fravel, 2011; Putra, 2020, 2023; Putra and Cangara, 2022; Yahuda, 2013). Consequently, ASEAN member states have remained cautious, with the majority of the states adopting what the international relations literature terms as a “hedging” policy vis-à-vis China's ascent (Goh, 2016; Haacke, 2019; Hiep, 2013; Kuik, 2008). A hedging position entails taking both defiance and deference measures (Kuik, 2021a; Kuik and Rozman, 2015) to ensure that ASEAN member states can garner economic benefits while maintaining some distance, avoiding an overly close attachment to China.

However, the newest member of ASEAN, Cambodia, has displayed some puzzling foreign policy gestures in the past several years. In 2012, during Cambodia's chairmanship of ASEAN, the ‘Phnom Penh Fiasco' resulted in the inability of ASEAN heads of state to collectively agree upon a joint communique (Minh Vu, 2019; Pich, 2021). Cambodia's Prime Minister at that time, Hun Sen, could not agree to include any mention of the developments taking place in the South China Sea in the communique (Chheang and Pheakdey, 2019; Jeldres, 2012; Pheakdey, 2012; Pich, 2021). For Cambodia, the South China Sea issue should be resolved and discussed through bilateral platforms, including between China and the Southeast Asian claimant states involved in the dispute. Unfortunately, a similar occurrence was observed at the 2016 ASEAN Summit, where Cambodia boldly expressed the need to refrain from discussing the South China Sea dispute on the regional platform (Bong, 2020; Mit, 2024). Cambodia's 2016 support for China's stance in the South China Sea disputes was met with a USD 600 million commitment from China to aid Cambodia (Po and Primiano, 2020).

Scholars have interpreted the growing alignment of the Cambodian and Chinese interests as “bandwagoning” with China, a position of siding with the ascending giant. There is an increasing presence of China in Cambodia, with BRI projects fueling extensive infrastructural development in Cambodia's cities (Lim, 2023; MOC, 2022; Nikkei, 2021; Ngin, 2022; Xinhua, 2018). As a result, discussions looking at Cambodia's alignment policies have primarily looked at Cambodia's foreign policy within the prism of realism's alignment and liberalism frameworks, concluding the bandwagoning of the country with China's interests due to economic interests (Cheunboran, 2021; Chheang, 2023; Doung et al., 2022; Pheakdey, 2012; Po and Primiano, 2020). Nevertheless, this opinion article argues that a sociological interpretation of Cambodia's actions in ASEAN is necessary to provide an alternative perspective to the alignment literature in international relations, which often misinterprets Cambodia's foreign policy. In doing so, this article bridges the social disorganization theory from sociology and criminology studies, starting from the original conception of the theory (as interpreted by the Chicago School) to recent findings that focus on the structural and communal origins of social disorganization. Through socio-psychological explanations, this opinion article explores the reasons why norm violations occur within ASEAN. The empirical investigation focuses on Cambodia's foreign policies in ASEAN from 2012 to 2022 and questions why Cambodia defied the ASEAN Way during its ASEAN Chairmanships. The following will first explain how the social disorganization theory will be utilized in this study.

## 2 Bridging the social disorganization theory into international relations

It has become a common occurrence to see sociological theories complement interpretations of studies in international relations. Wendt's “Social Theory of International Politics” in 1999 established the foundations by arguing that variables, such as the international system, structure, and anarchy, can be interpreted based on what states make of it (Wendt, 1999). Holsti's National Role Conception Theory also bridged sociological theory's role conception and interpreted how states interact based on the roles they embrace (Holsti, 1970). Constructivism's integration of sociological theories into international relations discourse has provided an alternative lens for interpreting realities, offering a more nuanced understanding (Baylis et al., 2019; Hopf, 1998, 2013; Karen and Tickner, 2020; Steans et al., 2010). Continuing this trend, studies have also attempted to bridge social psychology theories to understand better the foreign policy decisions made by states (Gildea, 2020; Kelman, 1997; Larson, 2017; Putra, 2024c; Rathbun, 2009). This opinion article will attempt to do the same with the social disorganization theory, building upon such trends.

Initially founded in the Chicago School through the works of Shaw and McKay in 1942, the social disorganization theory assesses the inability of communities to achieve common goals due to factors such as economic deprivation, residential mobility, and ethnic heterogeneity (Shaw and McKay, 1942). Explanations of the goals vary, but most scholars have agreed that establishing social controls is among the priorities when it comes to communal values and goals (Jensen, 2015; Sampson, 1985). The presence of the previously outlined factors is further exacerbated by other emerging factors, including industrialization and urbanization, which make it even more difficult for community organizations to assert control and monitor their residents' behaviors (Shaw and McKay, 1942).

A prominent theme within the theory is the development of delinquent behavior or criminal subcultures (Jensen, 2015; Papachristos and Zhao, 2015). As academics have argued, a disorganized society tends to lead to what Reiss explains as “behavior consequent to the failure of personal and social controls” (Reiss, 1951, p. 196). Considering the delinquent behaviors, studies have then attempted to explain the possible measures that can be taken to counter the lack of personal and social controls. Some ideas include establishing containment mechanisms, countering interests of criminal subcultures, and techniques to neutralize those thoughts (Briar and Piliavin, 1965; Matza, 1957; Reckless et al., 1956; Toby, 2017). In its current form, the social disorganization theory has evolved to initially explain crime and delinquency rates, assessing the internal and communal factors of delinquent behaviors (Benson et al., 2004; Browning, 2002; Morgan and Jasinski, 2016).

How, then, can social disorganization theory be bridged to the study of international relations? This study highlights the theory's core elements in concrete behaviors of states within the context of ASEAN. Discussions on delinquent subcultures and going against community values can be interpreted as an ASEAN state not abiding by the ASEAN Way and extending the influence of great powers within the regional group. The lack of social control that leads to delinquency can be attributed to the inability of ASEAN to counter foreign policies of non-neutrality shown by its member states. Meanwhile, the background factors leading to social disorganization, such as economic deprivation, are connected to a state's need for foreign investments, considering the unique contexts of Southeast Asian states as developing nations. The following section will explore these subthemes and bridge to a deeper understanding of the dynamics within ASEAN.

## 3 The social disorganization theory and Cambodia's ASEAN policies

This section provides a social disorganization theory's interpretation of Cambodia's ASEAN policies. It does not reference a specific thought or assumption within the theory and aims to establish links based on several central arguments made in the theory. This opinion article presents three key arguments: the presence of delinquent subcultures, the lack of social control, and the issue of income inequality, all of which impact Cambodia's ASEAN policies.

On the argument of delinquent subcultures, the sociological theory is that these behaviors lead to criminality. Bridged to Cambodia's ASEAN policies, delinquent subcultures can be interpreted as actions that go against the ASEAN Way. The ASEAN Way, understood as the communal values in place in the Southeast Asian region, was being undermined due to Cambodia's actions. Other Southeast Asian states' echoed the importance of including protests over the South China Sea developments in the 2012 joint communique. Cambodia decided not to mention the tensions and instead advocated for a bilateral approach (Po and Sims, 2022; Železný, 2022). Doing so violates the ASEAN Way in two ways. First, Cambodia's actions led to a lack of consensus-based decision-making processes within the organization. Second, it shows that China's growing influence on Cambodia has affected Cambodia's foreign policy in ASEAN, indicating that ASEAN is no longer immune to the non-interference of foreign powers (Poling et al., 2022; VOA, 2015).

During Cambodia's chairmanship in 2012, Cambodia's actions of defying the importance of including the South China Sea case in the joint communique impeded ASEAN's consensus-based decision-making processes (Po and Sims, 2022; Železný, 2022). In both the 2012 and 2016 incidents, in which Cambodia emphasized the importance of bilateral means of resolving the South China Sea, Cambodia's foreign policy, heavily influenced by China, indicates that ASEAN is no longer immune to the non-interference of foreign powers (Poling et al., 2022; VOA, 2015).

Making matters worse is that the delinquent subcultures displayed by Cambodia have evolved to support (in a limited sense) China's allies in Southeast Asia. Many of the claimant states in the South China Sea, located in Southeast Asia, have taken a more decisive stance vis-à-vis China (Blazevic, 2012; Putra, 2022; Qi, 2019; Raymond and Welch, 2022; Singh and Yamamoto, 2017). In contrast, some states that have faced democratic backsliding in past years, such as Cambodia, Laos, and Myanmar, have shown a greater leaning toward Beijing (Duangdee, 2021; Kobayashi and King, 2022; Myoe, 2015; Pang, 2017; Po and Primiano, 2020). Considering the ties between China and Myanmar, Cambodia decided to visit Myanmar in 2022 (Alexandra, 2024; HRW, 2022; Putra, 2024a).

The gesture was problematic in Myanmar for two reasons. First, a year before that, Myanmar faced backlash due to the military coup. Second, in 2022, Cambodia was the acting ASEAN chairman. ASEAN took the stance to not recognize Myanmar's military junta rule by issuing the “Five Points Consensus,” aiming to halt aid and demanding democratic changes to take place immediately (Muhammad et al., 2023; Oo and Tonnesson, 2023; Oponio Juris, 2022; Vasisht, 2024; Zaccaro, 2024). With Cambodia's Hun Sen visiting Myanmar in 2022, it gave the wrong impression that Cambodia was acting on behalf of ASEAN in making the official gesture.

The lack of social control to counter delinquent behaviors is equally vital in the social disorganization theory. As Sampson and Groves argued, social controls constrain the ability to generate delinquent behaviors (Sampson, 2012; Sampson and Groves, 1989). Discussion on social control focuses on the external or communal factors contributing to delinquent behaviors. Social control, in the context of Cambodia within ASEAN, refers to the organizational limitations on state actions. Unfortunately, the regional integration of ASEAN differs from that of organizations such as the European Union (EU). ASEAN is not a supranational body with the authority to impose mechanisms and regulations on its member states (Leifer, 1996; Snitwongse, 2007; Southgate, 2021; Yates, 2016). Therefore, ASEAN's ability to maintain the norms of non-interference, non-intervention, and a consensus-based decision-making process is determined by the ASEAN member states' willingness to uphold them.

As seen with Cambodia's actions since 2012, ASEAN did not have much social control over China's hierarchical relations with its member states. The only mechanism in place for ASEAN concerning the South China Sea was the existing negotiations of the Code of Conduct in the South China Sea (CoC; Mishra, 2017; Thayer, 2012). Besides that, no system within ASEAN is able to halt China's exertion of influence over ASEAN member states. The problem is that Cambodia has grown highly dependent on China's financial funding since the announcement of the BRI in 2013. Several notable transformations within the state can be seen in the reconstructed road and bridges, the establishment of the Sihanoukville Special Economic Zone (SSEZ), inter-city connections (including between the capital, Phnom Penh, and the SSEZ), airports, and harbors (Cheunboran, 2021; Chheang, 2023; Chong, 2017; Vannarith, 2019; Xinhua, 2023).

Therefore, it is not incorrect for existing studies to argue that Cambodia's representation of China's interests on regional platforms, such as ASEAN, constitutes a bandwagoning gesture with China. The sociological interpretation is that ASEAN, which relies on its member states to abide by the ASEAN way voluntarily, lacks social control to counter the impact of the hierarchical relations between China and Cambodia. The solutions, thus, were temporal. In 2012, Indonesia's then Foreign Affairs Minister, Marty Natalegawa, conducted “shuttle diplomacy” so that ASEAN would then issue a separate statement as an alternative to the joint communique (Emmers, 2014; Rattanasevee, 2014; Roberts and Widyaningsih, 2015). Meanwhile, following the 2016 incident, ASEAN's only recourse was to reiterate the importance of mutual trust and respect in accommodating differences in opinions (Parameswaran, 2016; Storey, 2018).

One of the central arguments in the social disorganization theory is that income inequality leads to delinquent behaviors. Scholars have argued that the disparate income categories within society hamper communication across different groups (Chamberlain and Hipp, 2015; Osgood and Chambers, 2000; Sampson, 1985; Wilson, 1987). Perhaps the most significant issue with ASEAN is economic disparity. For example, within ASEAN, this categorization is known as the CMLV (Cambodia, Myanmar, Laos, Vietnam). The CMLV represents the four newer members of ASEAN, which are less developed economically than the original members of the regional organization. The policy consequence is that, regionally, such as in the ASEAN-China Free Trade Agreement, the CMLV would be given particular preference through an extended adaptation period to adapt to the new economic measures in the region.

With the Southeast Asian economic disparity comes the consequence of a lack of communication between the more developed and less developing economies of ASEAN. The differences toward the common stance and communal values of ASEAN are thus the price paid. The importance of the South China Sea issue, for example, is not perceived by Cambodia in the same way as claimant states such as the Philippines and Vietnam (Basawantara, 2020; Chubb, 2022; Ha, 2019; Pemmaraju, 2016; Sangtam, 2021). Cambodia's perspective on the matter is that the representation of China and embracing the “ironclad friendship” (Po and Primiano, 2020) enables Cambodia to secure more substantial funding in the future. In other cases, Myanmar and Laos also exhibit similar attitudes of displaying deference measures toward China, albeit not to the same extent as Cambodia. One key factor is the increasing economic ties that have developed between Myanmar and Laos, which are openly dependent on the economic incentives and opportunities offered by China's BRI (Alatorre, 2024; Dossi and Gabusi, 2023; Kuik, 2021b; Kuik and Rosli, 2023). Out of the CMLV states, only Vietnam displays greater independence in its engagement with ASEAN, perhaps due to its interest in countering China's overlapping maritime claims. Therefore, a problem such as the South China Sea or the democratic backsliding in Myanmar does not fall under Cambodia's foreign policy concerns, leading to Cambodia's delinquent behavior vis-à-vis the ASEAN Way.
